# Antimicrobial Resistant Pathogens in the Oral Cavity of White (*Carcharodon carcharias*), Bull (*Carcharhinus leucas*) and Tiger (*Galeocerdo cuvier*) Sharks from the East Coast of Australia

**DOI:** 10.1007/s00284-025-04272-4

**Published:** 2025-05-21

**Authors:** Jessica McIntosh, Andrew Greenhill, Paul Butcher, Meagan Dewar

**Affiliations:** 1 Microbiology Group, Institute of Innovation, Science and Sustainability, Federation University Australia, Churchill, Australia; 2 Future Regions Research Centre, Federation University of Australia, Churchill, VIC Australia; 3 NSW Department of Primary Industries and Regional Development, National Marine Science Centre, Southern Cross University, Coffs Harbour, NSW 2450 Australia

## Abstract

Australia has one of the highest incidences of shark bites worldwide. Previous research on sharks has detected antibiotic-resistant pathogenic bacteria in the oral cavity, however, no such research has been conducted on species found in Australasian waters. In this study, 197 bacterial isolates were obtained from oral swabs taken from 153 sharks, including white (*Carcharodon carcharias*), tiger (*Galeocerdo cuvier*) and bull sharks (*Carcharhinus leucas*) caught on the east coast of Australia between May 2018 and April 2022. Isolates were identified using MALDI-TOF, and a disc diffusion assay was used to determine the antibiotic sensitivity of 72 isolates considered potential pathogens, including members of the genera *Pseudomonas, Vibrio, Aeromonas, Enterobacter, Klebsiella* and *Psychrobacter*. Resistance to one or more antibiotics was common, with 60%, 73%, and 74% of isolates from white, tiger and bull sharks resistant to one or more antibiotics, respectively. This research provides insights into which antibiotics are most suitable for treating shark bite wounds in eastern Australia, and possibly more broadly, to prevent infection. This study identified that over 51% of pathogens in the oral cavity of Australian sharks are resistant to one or more antibiotics, with most pathogens being resistant to commonly prescribed antibiotics. This research provides important information for medical professionals when it comes to treaty wounds inflicted by shark bites.

**Supplementary Information:**

The online version contains supplementary material available at 10.1007/s00284-025-04272-4.

## Introduction

Over the past 30 years the global number of shark bites has increased steadily from an average of ~ 180 per decade during 1950s–1980s, to an average of 650 between 1990 and 2010s [1]. The last decade (2010–2019) has seen a further increase in bites with 803 bites recorded, with an average fatality rate of 6% [1, 2]. Australia has the second highest incidence worldwide, accounting for ~ 20% of global shark bites. The largest proportion (40%) of shark bites in Australia occur in New South Wales [1].

Shark bite wounds are highly susceptible to infection, increasing the risk of mortality in victims [3]. Wound infection post shark bite is common and has been associated with the oral bacteria of sharks [3, 4]. Potentially pathogenic organisms have been detected in the oral cavity of sharks [4–8], and from the wounds of shark bite victims [9, 10]. Most research exploring the oral microbiota of sharks have been culture-based studies focussed on detecting pathogens of clinical importance. From these studies, bacterial species such as *Bacillus* spp., *Enterococcus* spp., *Psychrobacter* spp*., Salmonella enterica, Vibrio* spp. and *Pseudomonas* spp*.* have been identified [4, 8, 10, 11]. Recent 16S sequencing studies have found that the oral/teeth microbiota differs among species of sharks with the ecology of each shark species driving the inter-species differences in the microbial composition [11, 12]. From these studies several microbial taxa associated with causing infections from animal bites were identified including S*treptococcus*, *Staphylococcus*, *Corynebacterium*, *Enterococcus*, *Haemophilus, Vibrio*, *Salmonella enterica*, *Psychrobacter*, and *Halomonas* [11, 12]. These studies have demonstrated the diverse bacterial composition of the shark oral microbiota, and the variation between shark species and environments. Few studies have considered the oral bacteria of Australian sharks, with no research conducted on these populations in the last decade despite the increasing rate of shark attacks.

Current treatment of shark bite wounds varies but commonly involves prophylactic administration of antibiotics to reduce the risk of infection [4]. Antibiotic resistance has been detected in bacteria cultured from the oral cavity of sharks, although such studies are limited [4, 5]. Information available to Australian practitioners for antimicrobial therapy is limited to studies conducted overseas, with no data available on the oral microbiota of sharks in Australian waters. This lack of data could result in the administration of inappropriate antibiotics to bite victims [13]. With the lack of research into pathogens and associated antibiotic susceptibility from with sharks in Australia, where 20% of global shark attacks occur, it is unclear whether current treatment guidelines are optimal. The World Health Organization (WHO) endorses the avoidance of empirical treatment of infections whenever possible [14]. However, after some animal bites there may be a strong case for prophylaxis—such as shark bite wounds. Cooper et al. [15] suggest antibiotics that cover *Vibrio* spp. should be selected, with consideration also given to potential infection caused by *Staphylococcus* spp. and *Streptococcus* spp.

The three genera outlined above are recognised skin and soft tissue pathogens [16], however, other bacteria may be associated with shark bites. Recent studies have reinforced the need to reduce knowledge gaps on the broad range of potential pathogens that might be associated with shark bite wound infections [14]. This is particularly important for species inhabiting Australian waters given the lack of research, combined with a high burden of shark bites by global standards. This study aimed to identify potential human pathogens and quantify antimicrobial susceptibility of bacteria isolated from the oral cavities of white, tiger and bull sharks off eastern Australia using traditional culture techniques, MALDI-TOF analysis and antimicrobial disc diffusion assays. Those species were chosen as they are responsible for the majority of serious and fatal shark attacks in Australian waters.

## Materials and Methods

### Sample Collection

Samples were collected opportunistically between May 2018 and April 2022 from live white, bull and tiger sharks captured using SMART drumlines [17, 18] off the coast of New South Wales, Australia between Merimbula (− 36.887° S, 149.905° E), and Ballina (28.838° S, 153.562° E). Additional bull shark samples were collected from animals caught by hook and line from rivers between Karuah, NSW (28.838° S, 153.562° E) and Herbert River, Queensland (− 20.444° S, 148.693° E). Shark oral samples were collected using a customised collection apparatus consisting of an aluminium pole (40 cm in length, 5 cm wide and 2 cm deep) and stabilising clamp on the end to which a single swab was securely attached. After capture, each shark was secured to the starboard side of the vessel as part of a larger scientific study [19, 20] or brought into shallow water on the riverbank. For each shark, their head was raised out of the water and each swab rubbed along the posterior front top teeth and adjacent gum lines in a clockwise and then anti clockwise rotation 3 times, and then immediately stored in Amies medium (Global Scientific). Following collection, the swabs were stored at ambient room temperature and sent for analysis and the sharks were released.

### Culture and Identification

Swabs were inoculated on to blood agar plates and incubated at 35 °C for approximately 24 h. Isolates were sub-cultured to purity on Mueller Hinton agar, then diagnostic tests (Gram stains, oxidase, and catalase) were performed. The 400 isolates obtained were grouped based on basic biochemical test results and colony morphology, as well as shark species from which they were isolated. Isolates considered likely duplicate (same colony morphology, Gram reaction, catalase, and oxidase; and from the same individual or species of shark) were typically removed from further analyses, though some isolates with similar appearance were retained to determine diversity among similar colony morphologies. This led to 200 isolates being selected for identification by Matrix-Assisted Laser Desorption/Ionization-Time-of-Flight (MALDI-TOF) mass spectrometry and 16S RNA genome sequencing by Charles River Laboratories’ (Melbourne, Australia) Axcess® MALDI-TOF System (AccuPRO-ID). This system uses the Bruker MALDI Biotyper paired with Accugenix® databases. For isolates that could not be identified using MALDI-TOF, identification was done using Charles River Laboratories Bacterial ID Service Accugenix BacSeq (16S rRNA sequencing of 500 bp and identified using AccuGENX-ID database). Three isolates were unable to be cultured to purity, so were removed from further testing. All MALDI-TOF and BacSeq identification results are available in supplementary file 1.

### AMR Disc Diffusion Assays

Following identification, antibiotic susceptibility disc diffusion assays were conducted on recognised pathogenic organisms to determine the antibiotic resistance of each isolated bacterium, following Clinical and Laboratory Standards Institute (CLSI) guidelines [21], using *Escherichia coli* (ATCC 25922), *Staphylococcus aureus* (ATCC25923) and *Pseudomonas aeruginosa* (ATCC27853) as controls. Zones of inhibition were interpreted using data in the tables provided in the CLSI guidelines for the following organisms: Enterobacteriaceae, *Pseudomonas aeruginosa*, *Acinetobacter* (also used for *Aeromonas* spp.), *Staphylococcus* spp. (used for various non-fastidious gram positives, and *Enterococcus* spp. Antibiotics used for each group in this study are listed in Table 1. A full list of organisms and the specific cut-off zones used is provided in Supplementary Table 3.

**Table 1:** Antibiotics used for disc diffusion assay for each bacterial group

Antibiotic	Abbreviations	Bacterial group
Amoxicillin clavulanic acid (30 µg)	AMC30	Non-fastidious Gram positives
Ampicillin (10 µg)	AMP10	Enterobacteriaceae, *Acinetobacter*
Cefepime (30 µg)	FEP30	*Pseudomonas*, Enterobacteriaceae, *Acinetobacter/Aeromonas*
Cefoxitin (30 µg)	FOX30	Non-fastidious Gram positives
Ceftazidime (30 µg)	CAZ30	*Pseudomonas*, Enterobacteriaceae, *Acinetobacter/Aeromonas*
Chloramphenicol (30 µg)	C30	Enterobacteriaceae, *Acinetobacter/Aeromonas*, Non-fastidious Gram positives, *Enterococcus*
Ciprofloxacin (5 µg)	CIP5	*Pseudomonas*, Enterobacteriaceae, *Acinetobacter/Aeromonas*, Non-fastidious Gram positives, *Enterococcus*
Doxycycline (30 µg)	DO30	Enterobacteriaceae, *Acinetobacter*, Non-fastidious Gram positives, *Enterococcus*
Erythromycin (15 µg)	E15	Non-fastidious Gram positives, *Enterococcus*
Gentamicin (10 µg)	CN10	*Pseudomonas*, Enterobacteriaceae, *Acinetobacter/Aeromonas*
Imipenem (10 µg)	IMP10	Pseudomonads
Penicillin (10 µg)	P10	Non-fastidious Gram positives, *Enterococcus*
Piperacillin tazobactam (110 µg)	TZP110	*Pseudomonas*, *Acinetobacter/Aeromonas*
Tetracycline (30 µg)	TE30	Enterobacteriaceae, *Acinetobacter/Aeromonas*, Non-fastidious Gram positives, *Enterococcus*
Trimethoprim-sulfamethoxazole (25 µg)	SXT25	Enterobacteriaceae, *Acinetobacter/Aeromonas*, Non-fastidious Gram positives,
Vancomycin (30 µg)	VA30	*Enterococcus*

## Results

### Sample Collection

Swabs were collected from 51 white, 55 tiger and 78 bull sharks. These sharks ranged in total lengths (mean ± SD and range) between 235 ± 37.3 cm (167–300 cm), 226.2 ± 51 cm (128–365 cm), and 142.4 ± 59.6 cm (73–283 cm), for white, tiger and bull sharks, respectively.

### Cultured Organisms from Shark Teeth

A total of 90 bacterial species were detected from the 197 isolates identified using MALDI-TOF/16S sequencing (Supplementary Material Table X). Genera detected in at least two of the three target shark species are listed in Table 2. Of the 197 isolates identified, the most frequently encountered bacteria were *Pseudomonas* (13.7% of identified isolates), Psychrobacter spp. (9.1%), *Exiguobacterium spp.* (8.1%), *Rheinheimera* spp. (7.1%), *Enterobacter* spp. (6.1%) and *Aeromonas* spp. (4.6%). In white sharks (66 isolates) the most frequently encountered genera were *Rheinheimera* spp (11.9%), *Psychrobacter* spp (10.4%), *Exiguobacterium* spp (9.0%), *Pseudomonas* spp (9.0%), *Arenibacter* spp (7.5%) and *Carnobacterium* spp (6.0%). In tiger sharks (54 isolates), *Pseudomonas* spp (22.2%), *Psychrobacter* spp (13.0%), *Rheinheimera* spp (11.1%), *Acinetobacter* spp (5.6%), *Agrococcus* spp. (5.6%), *Exiguobacterium* spp. (5.6%), and *Microbacterium* spp. (5.6%) were common; whilst for bull sharks (78 isolates) *Enterobacter* spp. (11.5%), *Pseudomonas* spp. (11.5%), *Aeromonas* spp. (10.3%), *Exiguobacterium* spp. (10.3%), *Alcaligenes* spp. (9.0%), *Klebsiella* spp. (5.1%), and *Psychrobacter* spp. (5.1%) were common.

**Table 2:** Common genera of bacteria isolated from the oral cavity of multiple species of target sharks (bull, tiger and white sharks)

Genus	Number of isolates from each of the three shark species		
	White (*n* = 18)	Bull (*n* = 54)	Tiger (*n* = 22)
*Acinetobacter*	0	3	3
*Aeromonas*	1	8	0
*Arenibacter*	5	0	2
*Bacillus*	2	3	0
*Brachybacterium*	1	0	1
*Carnobacterium*	4	2	0
*Dietzia*	2	0	1
*Enterobacter*	2	9	1
*Escherichia*	0	2	1
*Exiguobacterium*	7	6	2
*Halomonas*	2	0	1
*Lysinibacillus*	2	2	0
*Macrococcus*	1	0	1
*Microbacterium*	1	0	3
*Micrococcus*	1	1	0
*Proteus*	2	3	1
*Pseudomonas*	6	9	13
*Psychrobacter*	7	4	7
*Rheinheimera*	8	0	6
*Shewanella*	2	2	2
*Staphylococcus*	1	0	1
*Stenotrophomonas*	0	2	1

### Antibiotic Susceptibility

**Fig. 1:**
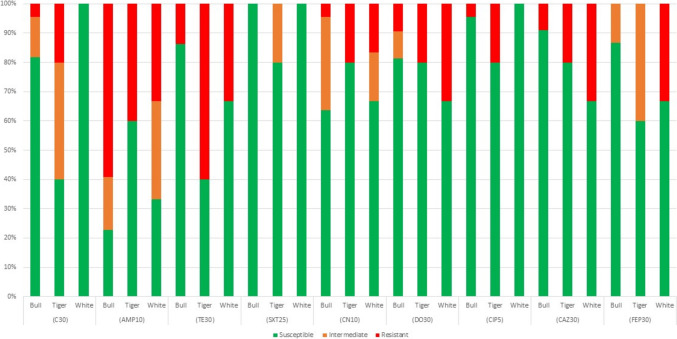
Proportion of Enterobacteriaceae isolates (*n* = 33) susceptible to selected antibiotics, using CLSI disk diffusion

Of the 72 isolates tested for antibiotic susceptibility (selected based on their potential to cause wound infection), 62.5% (45) of isolates were resistant to at least one antibiotic, whilst 36% (26) of isolates were resistant to more than one antibiotic. The percentage of bacteria isolated from white, bull and tiger sharks that were resistant to at least one antibiotic was 86%, 73%, and 42% of isolates, respectively. The proportion of bacteria that were resistant to two or more antibiotics was higher in white (53%) and bull (33%) sharks than tiger sharks (25%) (Supplementary Table 2). For Enterobacteriaceae species, 75.8% of isolates were resistant to one or more antibiotics, with 36% multi-drug resistant (2 or more). Resistance to ampicillin was most common (51.5% resistant, 18% intermediate resistance), followed by resistance to tetracycline (24%), doxycycline (18%), ceftazidime (15%) and gentamicin (9%) (Fig. 1).

**Fig. 2:**
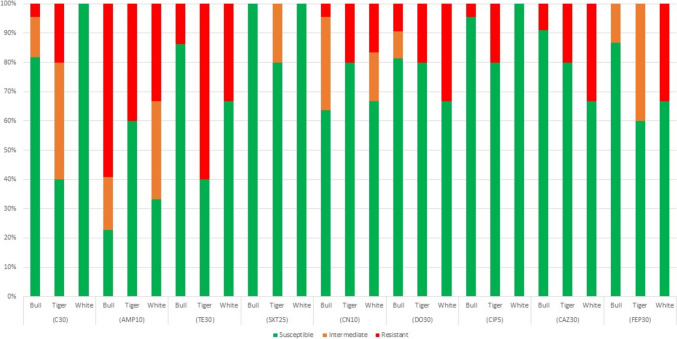
Proportion of *Pseudomonas* spp. isolates (*n* = 22) susceptible to selected antibiotics, using CLSI disk diffusion

**Fig. 3:**
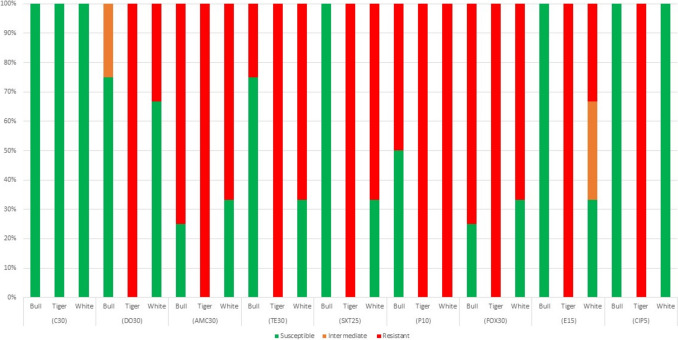
Proportion of non-fastidious gram-positive bacteria (*n* = 8) susceptible to selected antibiotics, using CLSI disk diffusion

For *Pseudomonas* spp, resistance was observed to ceftazidime (14% of isolates), cefepime (14%) and gentamicin (5%) (Fig. 2). For the small number of non-*Enterococcus* Gram positive bacteria isolated (*n* = 9) and identified in this study, resistance was observed to various antibiotics including amoxicillin-clavulanic acid (75% of isolates), penicillin (75%), cefoxitin (75%), tetracycline (50%), erythromycin (25%), doxycycline (25%), cotrimoxazole (12.5%), and ciprofloxacin (12.5%) (Fig. 3).

**Fig. 4:**
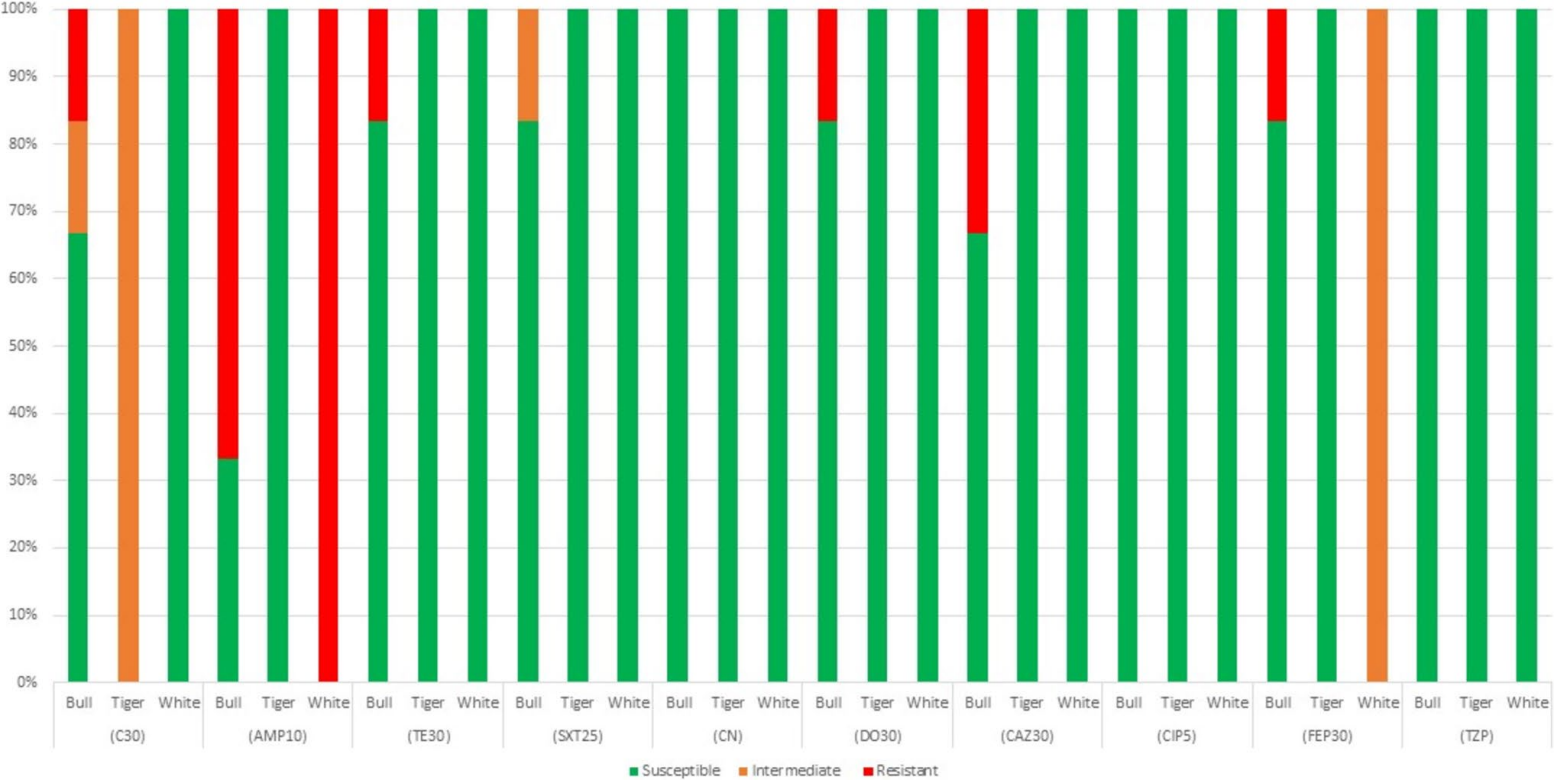
Proportion of Acinetobacter and Aeromonas isolates (*n* = 9) susceptible to selected antibiotics, using CLSI disk diffusion

For the *Acinetobacte*r (*n* = 5) and *Aeromonas* (*n* = 4) isolated and identified in this study, resistance was detected to ampicillin (55% of isolates), ceftazidime (22%) and 11% of isolates were resistant to each of chloramphenicol, tetracycline, doxycycline, and cefepime (Fig. 4). For *Enterococcus* spp. (*n* = 1), was susceptible to all antibiotics, except for Erythromycin which was intermediate.

## Discussion

Shark bite wounds commonly warrant prophylactic administration of antibiotics; however, the selection of antibiotics is based on limited data. This is the first study to detect potential pathogens of humans in the oral cavity of white, tiger and bull sharks in eastern Australia. Various pathogens associated with shark oral cavities and/or shark bite wounds in other global settings were detected in sharks sampled in this study, including *Pseudomonas* spp*., Staphylococcus* spp*., Bacillus* spp*. Vibrio parahaemolyticus* and *Micrococcus* spp. [4, 8]. In lieu of a geographically relevant review of the aetiology of shark bite wounds, this study of shark oral cavity microbes can help inform prophylactic treatment.

Several potential pathogens were detected that have not been previously reported in sharks or shark bite victims in Australia or overseas, including *Psychrobacter* spp*., Exiguobacterium* spp*., Shewanella algae,* and *Klebsiella* spp*.* The latter two genera (*Shewanella* and *Klebsiella*) are recognised human pathogens, capable of causing soft tissue infections [21–23]. Less frequently, some species of *Psychrobacter* (such as *P. arenosus*) and *Exiguobacterium* have the potential to cause wound infections and/or bacteremia in humans [24–26]. These two genera, and *Shewanella*, were detected across all three shark species and across multiple individuals. What role, if any, these bacteria play in shark bite wound infection remains unknown. We recognise that these pathogens are less commonly associated with wound infections than bacteria such as *Vibrio* spp. and *Staphylococcus* spp. However, with *Psychrobacter* and *Shewanella* known to be associated with infections following exposure to marine environments [27, 28], these organisms should be considered as potential aetiological agents in shark bite wound infections. 

This study intentionally applied a culture and identification approach to ensure we generated robust antimicrobial sensitivity data. Although antimicrobial resistance can be inferred from genomic data, culture and sensitivity remains the gold standard. In doing so we have gained an insight into the AMR characteristics of bacterial isolates associated with the shark oral cavity, but we have not sought to investigate all microbes (the microbiota or microbiome) present in the oral cavity of our target species of sharks.

There is a lack of consensus on which antibiotics, and which regimen, are best suited for use as prophylactics following a shark bite—various single and combination therapies having been recommended. Cooper et al. [15] suggest a combination of cephalexin and doxycycline, or cephalexin and ciprofloxacin. In one case report of a shark bite that occurred in Australia cephazolin, metronidazole and gentamicin were administered prophylactically [29]. The Center for Disease Control and Prevention (CDC) suggest that for *Vibrio vulnificus* infections (not specific to shark bites), a combination regimen is recommended including a 7–14-day course of doxycycline 100 mg twice daily and a third generation cephalosporin be administered (https://www.cdc.gov/vibrio/healthcare.html). While it is understandable that regimens will differ, these differences to some degree reflect the lack of data available to make informed decisions.

On the basis of our results, a prophylactic regimen that includes ciprofloxacin or gentamicin may be worth considering in Australia. Relative to other antibiotics tested, resistance to ciprofloxacin and gentamicin in Gram negative bacteria was low. The use of levofloxacin has been recommended for the treatment of shark bite wounds; however, third generation fluoroquinolones were not assayed in this study [8]. Given the relatively low levels of resistance to ciprofloxacin, these findings support the use of fluoroquinolones in the treatment and management of shark bite wound infections sustained in Australia. Future research should include levofloxacin or alternative third generation fluoroquinolone in antibiotic susceptibility testing.

Although only a small group of organisms, the Gram positive bacteria (Fig. 3, *n* = 8) were commonly resistant to beta-lactams such as penicillin, amoxicillin-clavulanic acid and cefoxitin. High levels of antibiotic resistance in Gram positive bacteria were observed for antibiotics except for chloramphenicol (100% susceptibility across all isolates). However, chloramphenicol is not a commonly used antibiotic, particularly in high-income countries, due to severe adverse effects. Where data on prophylactic antibiotics are available, cephalosporins are consistently used in shark bite treatments.

This study does not provide sufficient evidence to suggest a change in regimen away from cephalosporins; however, resistance to multiple antibiotics was detected in over 50% of isolates from white sharks and over 30% in bull sharks—including resistance to cephalosporins. The proportion of isolates resistant to multiple antibiotics in this study is higher than observed in previous studies in other global locations [4, 5, 8]. Currently, selection of an appropriate cephalosporin for use as a prophylactic is a difficult proposition. Further work may be required to look at a larger sample size and mechanisms of resistance.

The highest prevalence of resistance observed in target bacterial species was to commonly prescribed antibiotics such as ampicillin, trimethoprim-sulfamethoxazole, erythromycin, and tetracycline. Ampicillin was tested against all Gram negative, non-*Pseudomonas* spp. isolates, with between 40 and 100% of isolates from all species resistant. This was expected, given the high rates of resistance of many organisms to ampicillin in community and hospitals settings in Australia [30]. Furthermore, ampicillin resistance has previously been detected in marine environments, with resistant bacteria isolated from green sea turtles [31]. This finding reaffirms the choice of cephalosporins as prophylactic antibiotic of choice over penicillin-derived beta-lactams in shark bite wounds, though which cephalosporin is difficult to determine.

Previous studies considering antibiotic susceptibility of bacteria from the oral cavity of sharks are routinely used to guide empiric treatment of shark bites in Australia. These studies were conducted on multiple species of sharks across broad spatial scales including Bull, White, Tiger, Lemon (*Negaprion brevirostris*), Nurse (*Ginglymostoma cirratum*), Caribbean reef (*Carcharhinus perezii*), and Blacktip reef (*Carcharhinus melanopterus*) sharks [4–8, 11]. Interaminense et al. [8] concluded that gentamicin and vancomycin are the most effective choices of treatment for Gram positive cocci implicit in shark bite wound infections. Research conducted by Buck et al. [6] has been used over the last decade to guide treatment of Australian shark bite victims [32]. That research [6] was among the earliest studies to examine antibiotic susceptibility of shark oral bacteria, and recommended aminoglycosides as well as second and third generation cephalosporins. In our study, Aminoglycosides were represented by gentamicin with resistance present in bacteria isolated from white and tiger sharks. While there is still some merit in the recommendations of previous studies, antimicrobial resistance in bacteria isolated from sharks in the current study highlights that older guidelines/recommendations may not be appropriate for guiding treatment of shark bites in Australia, especially given the higher level of AMR in Australian sharks. Furthermore, while this study focussed on three species that are highly migratory [33–35], it was limited to sharks caught off eastern Australia. There could be spatial and temporal changes in the composition and proportions of bacterial microbiota in each shark species as they shift between latitudes and longitudes that cannot be accounted for in this study and may warrant further investigation.

Bacterial species isolated from bull (73%) and white (86%) sharks had a higher proportion of resistance to one or more antibiotics than those obtained from tiger sharks (42%). There are several explanations for the differences in the rate of AMR observed in bacterial species isolated from the different species. The presence of antibiotic resistance in marine microbes is believed to indicate multiple sources of exposure and transfer of resistant strains [36]. The presence of antibiotic-resistant bacteria is an indicator of pollution levels in surrounding environments [36]. The difference in resistance rates on bacteria between the species of sharks supports the assertion that migratory route and feeding grounds of marine animals exposes them to environmental pollutants which are potential sources of antibiotic-resistant bacteria [31, 37]. Pollutants such as heavy metals and polychlorinated biphenyl have been recently documented in sharks from the eastern Australia [38, 39], and this may impact on antimicrobial resistance. Furthermore, the diet and movements of white [33, 40, 41], tiger and bull sharks [34, 42–44] in Australia also differs between species. Therefore, it is plausible that the variations between species are driven by these biological factors.

Despite the findings of this study, and other literature supporting the presence of bacteria in the oral cavity of sharks, there are questions around the origin of bacteria detected. There is limited evidence to confirm that potential pathogens detected are unique to the teeth of sharks, and not the aquatic environment that the predator inhabits. Existing literature on bacteria from the oral cavity of sharks implicit in wound infections recognises the possibility that causative organisms arise from the aquatic environment and not the oral cavity or teeth of the sharks [6, 45]. Bacterial species that have been detected in the oral cavity, and isolated from shark inflicted wounds are commonly species that occur naturally in the marine environment. Pathogenic bacteria in bite wounds are most commonly reflective of the oral microbiota of the animal responsible for inflicting the injury, however they can be reflective of the environment in which the bite occurs [45].

This study identified potential pathogens that have in previous studies have been associated with causing skin and soft tissue infections in humans These bacterial pathogens have the potential to cause infection for shark bite victims in eastern Australia. Some organisms detected were consistent with the findings of previous research; however, this study identified the presence of microorganisms not previously associated with the oral cavity of sharks such as *Psychrobacter* spp. and *Exiguobacterium* spp.

Culture based studies have limitations, as they tend to target specific bacteria. Consequently, in this study we are unlikely to have detected the full diversity of potential pathogens present in the shark oral cavity. However, when investigating antimicrobial resistance, culture and sensitivity does have some advantages over metagenomic approaches. Future studies should incorporate molecular analysis of the microbiota of the shark oral cavity to better guide target species for culture and sensitivity. Further surveillance, potentially from other geographical locations in Australia, should be included in future studies if feasible. Empiric treatment of shark bite wounds in Australia based on findings of previous studies (conducted outside of Australia) should be approached with caution, due to the detection of resistance to antibiotics recommended in previous studies. Ciprofloxacin and gentamycin may be of value, but which cephalosporins are of most benefit to the patient remains unclear.

## Supplementary Information

Below is the link to the electronic supplementary material.

Supplementary file1 (DOCX 60 kb)

## Data Availability

Data are available in the attached supplementary documents.
