# Supplementary material for: Antimicrobial Resistant Pathogens in the Oral Cavity of White (Carcharodon carcharias), Bull (Carcharhinus leucas) and Tiger (Galeocerdo cuvier) Sharks from the East Coast of Australia
Source: Curr Microbiol. 2025 May 21;82(7):300. doi: 10.1007/s00284-025-04272-4 (PMC12095389; doi:10.1007/s00284-025-04272-4)
Supplement: Supplementary file 1 — Supplementary file1 (DOCX 60 kb) [file 284_2025_4272_MOESM1_ESM.docx]

Supplementary Table 1. Bacterial species isolated from each shark species

| **Genus** | **Species** | **# of Isolates per shark species** | | |
| --- | --- | --- | --- | --- |
|  |  | **White** | **Bull** | **Tiger** |
| *Achromobacter* | *Denitrificans/insolitus/piechaudii/ruhlandii/spanius/ xylosoxidans* |  | 1 |  |
| *Acinetobacter* | *calcoaceticus* |  | 1 | 2 |
|  | *lactucae* |  | 1 |  |
|  | *venetianus* |  | 1 | 1 |
| *Aerococcus* | *urinaeequi/viridans* | 1 |  |  |
| *Aeromonas* | *caviae / enteropelogenes* |  | 1 |  |
|  | *hydrophila* | 1 | 3 |  |
|  | *popoffii* |  | 1 |  |
|  | *punctata* |  | 2 |  |
|  | *veronii* |  | 1 |  |
| *Agrococcus* | *baldri* |  |  | 1 |
|  | *citreus* |  |  | 2 |
| *Alcaligenes* | *faecalis* |  | 7 |  |
| *Arenibacter* | *latericius* | 5 |  | 2 |
| *Arthrobacter (Glutamicibacter)* | *creatinolyticus* | 1 |  |  |
| *Bacillus* | *altitudinis/pumilus/safensis* |  | 1 |  |
|  | *cereus group* | 2 | 2 |  |
| *Brachybacterium* | *conglomeratum/paraconglomeratum* | 1 |  |  |
|  | *massiliense* |  |  | 1 |
| *Carnobacterium* | *maltaromaticum* | 4 | 2 |  |
| *Citrobacter* | *amalonaticus/farmeri* |  | 2 |  |
|  | *freundii complex* |  | 1 |  |
| *Dietzia* | *cercidiphylli/natronolimnaea/psychralcaliphila* |  |  | 1 |
|  | *kunjamensis/maris/schimae* | 2 |  |  |
| *Enterobacter* | *cloacae complex* | 2 | 9 | 1 |
| *Enterococcus* | *faecalis* | 1 |  |  |
| *Escherichia* | *coli/fergusonii/Shigella boydii/dysenteriae/flexneri/sonnei* |  |  | 1 |
|  | *hermannii* |  | 2 |  |
| *Exiguobacterium* | *acetylicum* | 1 |  | 1 |
|  | *aurantiacum* | 1 |  |  |
|  | *profundum* | 1 | 6 | 1 |
|  | *undae* | 4 |  |  |
| *Halomonas* | *meridiana* | 2 |  | 1 |
| *Klebsiella* | *pneumoniae* |  | 2 |  |
|  | *variicola* |  | 2 |  |
| *Kurthia* | *Gibsonii* |  | 1 |  |
| *Lelliottia* | *amnigena* |  |  | 1 |
| *Lysinibacillus* | *boronitolerans/xylanilyticus* | 1 |  |  |
|  | *fusiformis* | 1 | 2 |  |
| *Macrococcus* | *canis/caseolyticus* | 1 |  |  |
| *Marinobacter* | *litoralis* |  |  | 1 |
|  | *maritimus* | 1 |  |  |
| *Microbacterium* | *esteraromaticum* |  |  | 1 |
|  | *hydrocarbonoxydans* |  |  | 1 |
|  | *liquefaciens/maritypicum/oxydans* | 1 |  | 1 |
| *Micrococcus* | *antarcticus* | 1 |  |  |
|  | *luteus* |  | 1 |  |
| *Morganella* | *morganii* |  | 2 |  |
| *Ochrobactrum* | *anthropic* | 1 |  |  |
| *Oerskovia* | *enterophila* |  | 1 |  |
|  | *turbata* |  | 1 |  |
| *Proteus* | *hauseri/penneri/vulgaris* | 2 | 2 | 1 |
|  | *mirabilis* |  | 1 |  |
| *Providencia* | *rettgeri* |  |  | 2 |
| *Pseudomonas* | *anguilliseptica* | 1 |  |  |
|  | *fluorescens group* | 2 | 1 | 2 |
|  | *granadensis / koreensis / moraviensis* | 1 |  |  |
|  | *japonica* |  |  | 1 |
|  | *laurylsulfatophila* | 1 |  |  |
|  | *mendocina* |  | 3 | 2 |
|  | *oryzihabitans* |  |  | 1 |
|  | *perfectomarina* |  | 1 |  |
|  | *putida group* | 1 | 3 | 1 |
|  | *sediminis* |  | 1 | 1 |
|  | *segetis* |  |  | 1 |
|  | *xanthomarina* |  |  | 2 |
|  | species (non-speciated) |  |  | 2 |
| *Psychrobacter* | species (non-speciated) | 2 |  |  |
|  | *arenosus* | 2 |  |  |
|  | *celer* | 1 | 2 | 5 |
|  | *faecalis* | 1 |  | 1 |
|  | *maritimus* | 1 | 2 | 1 |
| *Rheinheimera* | *baltica* |  |  | 1 |
|  | *pacifica* |  |  | 1 |
|  | *salexigens* | 8 |  | 4 |
| *Rhodococcus* | *biphenylivorans* | 2 |  |  |
| *Rossellomorea* | *vietnamensis* |  |  | 1 |
| *Sanguibacter* | *inulinus* | 2 |  |  |
| *Serratia* | *marcescens/nematodiphila/ureilytica* | 1 |  |  |
| *Shewanella* | *algae* |  | 2 |  |
|  | *algae / haliotis* |  |  | 1 |
|  | *baltica* | 2 |  |  |
|  | *hafniensis* |  |  | 1 |
| *Staphylococcus* | *capitis* | 1 |  |  |
|  | *haemolyticus* |  |  | 1 |
| *Stenotrophomonas* | *maltophilia* |  | 2 |  |
|  | *rhizophila* |  |  | 1 |
| *Vibrio* | *parahaemolyticus* |  | 1 |  |

**Supplementary Table 2. Level of Resistance for Isolates**

| **Isolate** | **Bacterial Genus** | **Bacterial Species** | **Species** | **# of Antibiotics resistant to** |
| --- | --- | --- | --- | --- |
| 94B-a | Pseudomonas | fluorescens group | Bull | 0 |
| 108B-b | Pseudomonas | mendocina | Bull | 0 |
| 114B | Pseudomonas | perfectomarina | Bull | 0 |
| 120B | Micrococcus | luteus | Bull | 0 |
| NL023-b | Morganella | morganii | Bull | 0 |
| NL031-a | Alcaligenes | faecalis | Bull | 0 |
| NL032-a | Alcaligenes | faecalis | Bull | 0 |
| NL032-b | Alcaligenes | faecalis | Bull | 0 |
| NL033-a | Alcaligenes | faecalis | Bull | 0 |
| NL033-b | Alcaligenes | faecalis | Bull | 0 |
| NL034-a | Enterobacter | cloacae complex | Bull | 0 |
| NL034-b (1) | Enterobacter | cloacae complex | Bull | 0 |
| NL053-b | Pseudomonas | mendocina | Bull | 0 |
| NL115-b | Alcaligenes | faecalis | Bull | 0 |
| NL146 | Alcaligenes | faecalis | Bull | 0 |
| NL176 | Citrobacter | freundii complex | Bull | 0 |
| 171T-b | Pseudomonas | fluorescens group | Tiger | 0 |
| 187T-b | Pseudomonas | oryzihabitans | Tiger | 0 |
| 201T-b | Pseudomonas | mendocina | Tiger | 0 |
| 210T-a | Pseudomonas sp. | Species | Tiger | 0 |
| 216T-a | Acinetobacter | calcoaceticus | Tiger | 0 |
| 216T-b | Acinetobacter | calcoaceticus | Tiger | 0 |
| 219T-a | Pseudomonas | japonica | Tiger | 0 |
| 285W-2 | Proteus | hauseri/penneri/vulgaris | White | 0 |
| 653W-b | Enterococcus | faecalis | White | 0 |
| 110B | Enterobacter | cloacae complex | Bull | 1 |
| 111B | Enterobacter | cloacae complex | Bull | 1 |
| 121B | Acinetobacter | venetianus | Bull | 1 |
| FN20 | Klebsiella | variicola | Bull | 1 |
| FN23 | Enterobacter | cloacae complex | Bull | 1 |
| FN24-a | Enterobacter | cloacae complex | Bull | 1 |
| NL131-b | Escherichia | hermannii | Bull | 1 |
| NL144 | Klebsiella | pneumoniae | Bull | 1 |
| NL150-a | Citrobacter | amalonaticus/farmeri | Bull | 1 |
| NL161 | Citrobacter | amalonaticus/farmeri | Bull | 1 |
| NL174-a | Klebsiella | pneumoniae | Bull | 1 |
| NL27-b | Aeromonas | hydrophila | Bull | 1 |
| NL46-a | Enterobacter | cloacae complex | Bull | 1 |
| 202T | Enterobacter | cloacae complex | Tiger | 1 |
| 213T-a | Providencia | rettgeri | Tiger | 1 |
| 127W-a | Aeromonas | hydrophila | White | 1 |
| 304W | Pseudomonas | putida group | White | 1 |
| 615W-b | Staphylococcus | capitis | White | 1 |
| 104B-b | Enterobacter | cloacae complex | Bull | 2 |
| 116B-c | Aeromonas | punctata | Bull | 2 |
| 123B-c | Shewanella | algae | Bull | 2 |
| FN18 | Shewanella | algae | Bull | 2 |
| NL025-b | Aeromonas | veronii | Bull | 2 |
| NL053-a | Acinetobacter | lactucae | Bull | 2 |
| NL120-c | Bacillus | altitudinis/pumilus/safensis | Bull | 2 |
| NL147-a | Proteus | mirabilis | Bull | 2 |
| NL150-b | Klebsiella | variicola | Bull | 2 |
| NL27-a | Acinetobacter | calcoaceticus | Bull | 2 |
| NL51-a | Pseudomonas | putida group | Bull | 2 |
| 180T | Escherichia | coli/fergusonii/Shigella boydii/dysenteriae/flexneri/sonnei | Tiger | 2 |
| 624W-a2 | Pseudomonas | laurylsulfatophila | White | 2 |
| NL113-b | Bacillus | cereus group | Bull | 3 |
| NL148 | Proteus | hauseri/penneri/vulgaris | Bull | 3 |
| 207T | Providencia | rettgeri | Tiger | 3 |
| 213T-b | Proteus | hauseri/penneri/vulgaris | Tiger | 3 |
| 105WA | Proteus | hauseri/penneri/vulgaris | White | 3 |
| 127W-b | Serratia | marcescens/nematodiphila/ureilytica | White | 3 |
| 654W-a | Pseudomonas | fluorescens group | White | 3 |
| 663W-a | Enterobacter | cloacae complex | White | 3 |
| 663W-b | Enterobacter | cloacae complex | White | 3 |
| NL117-b | Bacillus | cereus group | Bull | 4 |
| 289W-1 | Bacillus | cereus group | White | 4 |
| 624W-a1 | Shewanella | baltica | White | 5 |
| 634W | Bacillus | cereus group | White | 6 |
| 167T-b | Staphylococcus | haemolyticus | Tiger | 8 |
|  |  |  |  |  |
|  | Highly unlikely to cause skin and soft tissues infection in humans (but may cause infection in other organ systems, and/or other members of the genus are skin pathogens). | |  |  |
|  | Rarely causes and/or opportunists cause of skin and soft tissue infections. | |  |  |
|  | Recognised skin and soft tissue pathogen in humans. | |  |  |

**Supplementary Table 3. Cut-off zones for antimicrobial disc diffusion**

| ***Staphylococcus*** | **Susceptible** | **Intermediate** | **Resistant** | **Source** |
| --- | --- | --- | --- | --- |
| Chloramphenicol (30ug) (C30) | ≥18 | 13-17 | ≤12 | M100 33rd ed (2023) |
| Penicillin (10ug) (P10) | ≥29 | - | ≤28 | M100 26th ed (2016) |
| Amoxicillin clavulanic acid (AMC30) | ≥20 | - | ≤19 | M100 22nd ed (2012) |
| Tetracycline (30ug) (T30) | ≥19 | 15-18 | ≤14 | M100 33rd ed (2023) |
| Erythromycin (15ug) (E15) | ≥23 | 14-22 | ≤13 | M100 33rd ed (2023) |
| Trimethoprim-sulfamethoxazole (SXT25) | ≥16 | 11-15 | ≤10 | M100 33rd ed (2023) |
|  |  |  |  |  |
| ***Enterococcus*** | **Susceptible** | **Intermediate** | **Resistant** |  |
| Chloramphenicol (30ug) (C30) | >18 | 13-17 | <12 | M100 33rd ed (2023) |
| Penicillin (10ug) (P10) | >15 | - | <14 | M100 33rd ed (2023) |
| Tetracycline (30ug) (T30) | >19 | 15-18 | <14 | M100 33rd ed (2023) |
| Erythromycin (15ug) (E15) | >23 | 14-22 | <13 | M100 33rd ed (2023) |
|  |  |  |  |  |
| ***Pseudomonas*** | **Susceptible** | **Intermediate** | **Resistant** |  |
| Ceftazidime 30 ug (CAZ) | >18 | 15-17 | <14 | M100 33rd ed (2023) |
| Ciprofloxacin 5 ug (CIPS) | >21 | 16-20 | <15 | M100 28th ed (2018) |
| Gentamicin 10 ug (CN) | >15 | 13-14 | <12 | M100 28th ed (2018) |
| Imipenem 10 ug (IMP) | >19 | 16-18 | <15 | M100 33rd ed (2023) |
| Piperacillin tazobactam 110 ug (TZP) | >22 | 18-21 | <17 | M100 33rd ed (2023) |
|  |  |  |  |  |
| **Enterobacteriaceae** | **Susceptible** | **Intermediate** | **Resistant** |  |
| Chloramphenicol (30ug) (C30) | >18 | 13-17 | <12 | M100 33rd ed (2023) |
| Ampicillin (10ug) | >17 | 14-16 | <13 | M100 33rd ed (2023) |
| Tetracycline (30ug) | >15 | 12 -14 | <11 | M100 33rd ed (2023) |
| rimethoprim-sulfamethoxazole (SXT25) | >16 | 11-15 | <10 | M100 33rd ed (2023) |
| Gentamicin (G10) | >18 | 15-17 | <14 | M100 33rd ed (2023) |
| Ceftazidime (30ug) (CAZ) | >21 | 18-20 | <17 | M100 33rd ed (2023) |
|  |  |  |  |  |
| **Acinetobacter** | **Susceptible** | **Intermediate** | **Resistant** |  |
| Chloramphenicol (30ug) (C30) | >18 | 13-17 | <12 | M100 33rd ed (2023) |
| Ampicillin (10ug) | >15 | 12-14 | <11 | M100 33rd ed (2023) |
| Tetracycline (30ug) | >15 | 12-14 | <11 | M100 33rd ed (2023) |
| Trimethoprim-sulfamethoxazole (SXT25) | >16 | 11-15 | <10 | M100 33rd ed (2023) |
| Gentamicin (G10) | >15 | 13-14 | <12 | M100 33rd ed (2023) |
| Ceftazidime (30ug) (CAZ) | >18 | 15-17 | <14 | M100 33rd ed (2023) |
